# Predictive value of low serum interleukin-33 levels in acute ischemic stroke outcomes

**DOI:** 10.3389/fneur.2024.1503443

**Published:** 2024-11-22

**Authors:** Wei Liu, Dongliang Luo, Xingyu Liu, Yuqing Zhang, Zhong Wang

**Affiliations:** ^1^Department of Neurosurgery, The First Affiliated Hospital of Soochow University, Suzhou, China; ^2^Department of Neurosurgery, Affiliated Hospital of Shandong Second Medical University, Weifang, Shandong, China

**Keywords:** ischemic stroke, prognosis, biomarker, IL-33, Chinese

## Abstract

**Background:**

Human interleukin-33 (IL-33), a member of the IL-1 family, has been identified as a therapeutic target due to its role as a proinflammatory mediator in various diseases. This study aims to evaluate the prognostic value of serum IL-33 levels in patients admitted with their first-ever acute ischemic stroke.

**Methods:**

This single-center, prospective, observational study included 216 patients with acute ischemic stroke. Serum IL-33 levels were measured at hospital admission to assess their predictive value for functional outcomes and mortality within 3 months. IL-33 levels were dichotomized at the median into two groups: the reduced group (IL-33 ≤ median) and the normal group (IL-33 > median).

**Results:**

The median age of the 216 patients was 66 years (interquartile range [IQR], 56–75), with 132 (61.6%) being women. IL-33 serum levels were inversely correlated with stroke severity, as measured by the National Institutes of Health Stroke Scale (NIHSS) score and lesion size. Patients in the reduced IL-33 group had a higher rate of unfavorable outcomes (55.6% vs. 18.5%; absolute difference, 29.2% [95% confidence interval (CI), 24.5% to 34.4%]; odds ratio (OR), 3.19 [95% CI, 1.72 to 5.91]) and mortality (24.1% vs. 3.7%; absolute difference, 15.8% [95% CI, 13.1% to 18.3%]; OR, 4.12 [95% CI, 1.38 to 12.31]) compared to the normal group. Furthermore, IL-33 levels enhanced the prognostic accuracy of the NIHSS for predicting functional outcomes (combined area under the curve [AUC], 0.84; 95% CI, 0.79–0.84; *P* < 0.001) and mortality (combined AUC, 0.88; 95% CI, 0.83–0.94; *P* < 0.001).

**Conclusion:**

This study demonstrates that lower IL-33 levels are associated with increased stroke severity and poorer prognosis. These findings suggest that IL-33 may serve as a valuable biomarker for predicting poor outcomes following acute ischemic stroke.

## Introduction

Stroke remains a leading cause of mortality and long-term physical and cognitive disability in China, with over 2 million new cases each year (1, 2). According to the National Epidemiological Survey of Stroke in China (NESS-China), there were approximately 11 million prevalent stroke cases, 2.4 million new strokes, and 1.1 million stroke-related deaths in 2013 (3). These statistics underscore the urgent need for enhanced stroke prevention and management strategies in China (4).

Interleukin-33 (IL-33), a member of the IL-1 family, functions as an endogenous alarmin released by damaged or necrotic barrier cells, such as endothelial and epithelial cells, during homeostasis and inflammation (5). IL-33 is a therapeutic target due to its role as a proinflammatory mediator in various diseases (6), including allergic airway inflammation (7), anaphylaxis (8), and autoimmune diseases (9). Additionally, IL-33 has been shown to reduce atherosclerosis in animal models (10) and protect against cardiac dysfunction in mechanically overloaded hearts (11). Li et al. (12) demonstrated that IL-33 protects mice from abdominal aortic aneurysm formation by enhancing ST2-dependent regulatory T-cell expansion and their immunosuppressive activities, suggesting that IL-33 signaling possesses both proinflammatory and cardioprotective properties (13). Nuclear IL-33 functions as a stored alarmin that is released when barriers are breached (14).

In the context of central nervous system diseases, IL-33/ST2 signaling plays a dual role, impacting conditions such as neurodegenerative diseases (15), cerebrovascular diseases (16), traumatic CNS injury (17), and chronic pain (18). Previous studies have demonstrated that elevated serum IL-33 concentrations are associated with inflammation, disease severity, and prognosis in conditions such as traumatic brain injury (19) and aneurysmal subarachnoid hemorrhage (20). Liu et al. found that serum IL-33 levels increase with infarct size, suggesting IL-33's role in the pathogenesis and progression of acute cerebral infarction (21). Despite these observations, the mechanisms underlying IL-33 release remain poorly understood. Chen et al. further identified low serum IL-33 as an independent predictive biomarker for hemorrhagic transformation and adverse outcomes in acute ischemic stroke (22).

These findings underscore the importance of exploring the relationship between IL-33 levels and clinical severity or prognosis in stroke patients. To address this, we assessed the prognostic value of serum IL-33 at the time of hospital admission in a cohort of 216 patients experiencing their first-ever acute ischemic stroke (AIS).

## Materials and methods

### Study design and setting

This prospective observational cohort study was conducted at the Affiliated hospital of Shandong Second Medical University. Between December 2018 and February 2020, we included consecutive patients who were admitted with their first-ever acute ischemic stroke. We obtained written informed consent from either the patient or a relative. The study was conducted in accordance with the Declaration of Helsinki (1964) and adhered to the consolidated standards for reporting observational trials (23). Approval was obtained from the Ethics Committee of the Affiliated hospital of Shandong Second Medical University.

### Patients and clinical variables

Patients admitted with acute ischemic stroke within 48 h of symptom onset, as defined by World Health Organization criteria (24), were included in the analysis. Within the first 24 h after admission, we collected data on age, sex, BMI, blood pressure, body temperature, and traditional risk factors including smoking and drinking habits, history of hypertension, diabetes, hypercholesterolemia, coronary heart disease, atrial fibrillation, and family history of cardiovascular events. We also documented pre-stroke medications—oral anticoagulants, antiplatelet agents, antihypertensives, and statins—and acute treatments like IV thrombolysis and mechanical thrombectomy. Stroke severity was evaluated using the NIH Stroke Scale (25), and etiology was classified according to TOAST criteria (26), identifying the primary causes as large-artery arteriosclerosis, cardioembolism, small-artery occlusion, or other undetermined etiologies.

Within the first 24 h after admission, cranial computed tomography (CT) and/or magnetic resonance imaging (MRI) were performed on all patients to rule out intracranial hemorrhage and confirm the diagnosis of ischemic stroke. MRI with diffusion-weighted imaging (DWI) was available for some patients. Lesion sizes were quantified using a semi-quantitative method (27) and categorized into three groups to reflect typical stroke patterns: small lesions (< 10 mL), medium lesions (10–100 mL), and large lesions (>100 mL) (28).

### Laboratory analysis

Fasting blood samples were collected and processed within the first 24 h after admission. Blood was centrifuged at 4°C for 15 min at 1,000 x g. Serum was then separated and stored at−80°C. Serum IL-33 levels were measured using a Human IL-33 ELISA Kit (No. ab119547; ABCAM, LTD, Shanghai, China), with a detection sensitivity of 0.2 pg/ml and a range of 7.8 to 500 pg/ml. The intra-assay and inter-assay coefficients of variation were 4.0–4.7% and 6.0–6.9%, respectively, with a sample recovery rate of 67% to 79%. Additional biomarkers, including C-reactive protein (CRP), glucose (GLU), and homocysteine (HCY), were also measured using an enzyme cycling method with the BS800M analyzer (MINDRAY, Shenzhen, China). Values below the detection limit were recorded as the lower limit of detection.

### Follow-up and outcomes

Three months after admission, all discharged survivors underwent a structured telephone interview as part of the follow-up. The primary endpoint was a favorable functional outcome, defined as a score of 0 to 2 on the modified Rankin Scale (mRS) (29). The secondary endpoint was mortality from any cause within the 3-month follow-up period. Outcome assessments were conducted by two trained medical students, who were blinded to the patients' IL-33 levels, via telephone interviews with either the patient or, if necessary, the closest relative.

### Statistical analysis

Data were summarized as counts (percentage) for categorical variables and medians (interquartile ranges [IQRs]) for continuous variables. Continuous variables were compared between groups using the Mann–Whitney U test for two-group comparisons or the Kruskal–Wallis one-way analysis for multigroup comparisons. Categorical variables were compared using the Chi-square test.

The association between IL-33 levels and stroke severity was assessed using Spearman's rank correlation with NIHSS scores and lesion size. NIHSS scores were categorized into minor stroke (NIHSS ≤ 5), moderate (NIHSS 6–10), and high clinical severity (NIHSS > 10) for further analysis.

Statistical analyses were conducted to evaluate the predictive ability of IL-33 for functional outcomes and mortality at 3 months. Logistic regression models were used to examine the relationship between IL-33 levels—categorized by median values as reduced (≤ median) or normal (> median)—and the two endpoints, with results reported as odds ratios (ORs) along with 95% confidence intervals (CIs). Both unadjusted and multivariate models, accounting for significant predictors, were applied.

The predictive models' discrimination was assessed using receiver operating characteristic (ROC) curve analysis, with results reported as the area under the curve (AUC). The improvement in score performance by adding IL-33 to NIHSS was evaluated using nested logistic regression models. We employed a reclassification model to further assess the added benefit of IL-33 levels compared to the NIHSS score alone in risk prediction. For net reclassification improvement, only changes in estimated prediction probabilities that resulted in a shift from one risk category to another were considered.

For mortality prediction, Kaplan–Meier survival curves stratified by median IL-33 levels were calculated. All statistical tests were two-tailed with a significance level of *P* < 0.05. Analyses were performed using GraphPad Prism (version 5.0), R version 2.8.1 with the ROCR package (version 1.0–2), and Stata 9.2.

### Data available

Please contact the corresponding author for the data request.

## Results

### Patients

**Figure 1:**
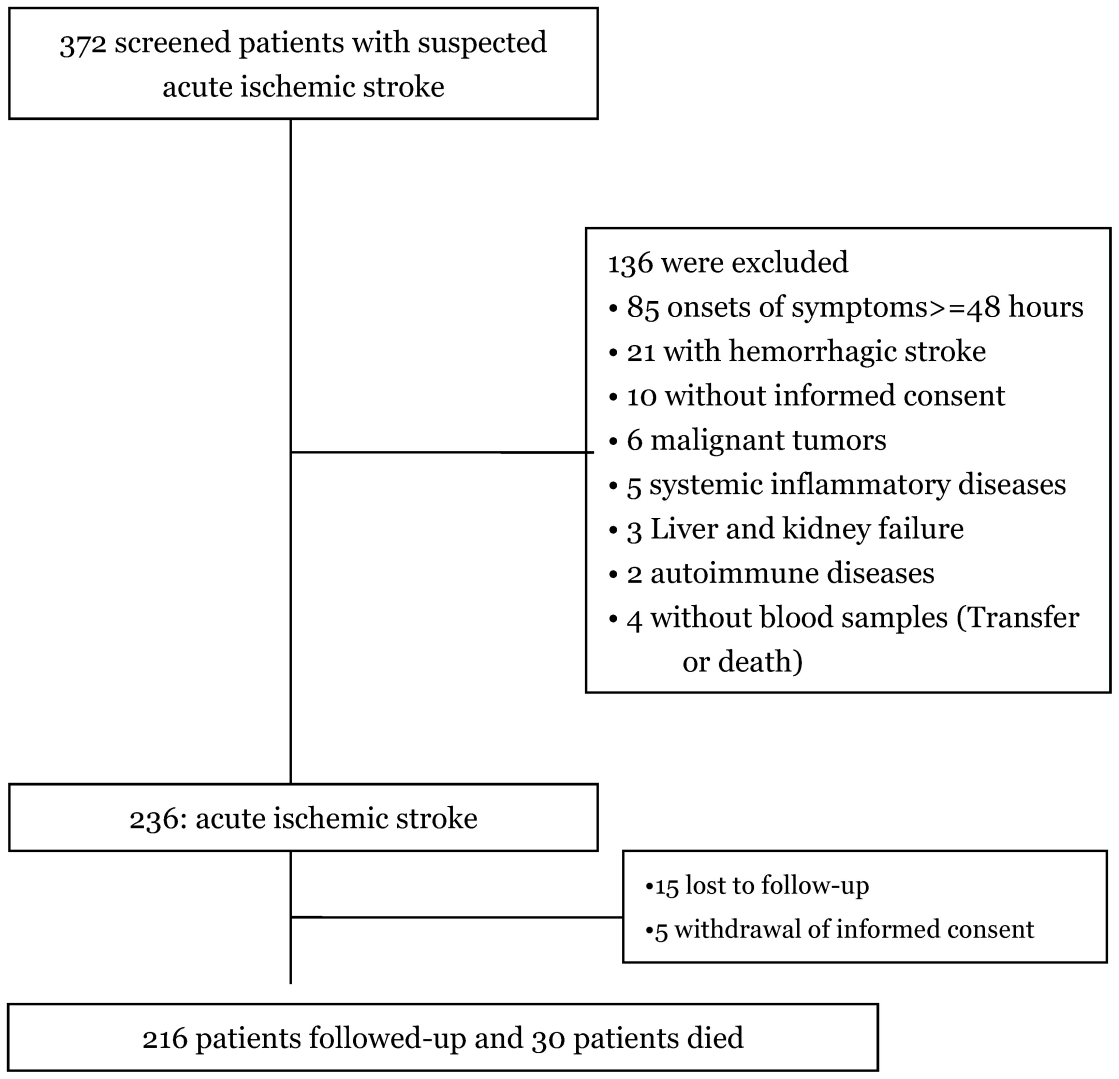
Study profile/flow sheet of the study.

Among the 372 patients initially screened for suspected first-ever acute ischemic stroke, 236 were confirmed with the diagnosis, and 216 completed follow-up and were included in the final analysis (Figure 1). The baseline characteristics of these 216 patients—including age (*P* = 0.12), gender (*P* = 0.93), BMI (*P* = 0.44), and NIHSS scores (*P* = 0.35)—were similar to those of the overall cohort of ischemic stroke patients.

### Baseline characteristics of the study population

The included patients had a median age of 66 years (IQR, 56–75), with 132 (61.6%) being women. The median systolic and diastolic blood pressures were 155 mmHg (IQR, 130–175) and 95 mmHg (IQR, 85–105), respectively. Among them, 155 (71.8%) were diagnosed with hypertension, 59 (27.3%) with diabetes mellitus, 20 (9.3%) had atrial fibrillation, 12 (5.6%) reported a family history of stroke, and 46 (21.3%) were smokers. Upon admission, the median NIHSS score was 7 (IQR, 4–12), and 24 patients (11.1%) received IV thrombolysis. The median serum IL-33 level was 52.6 pg/ml (IQR, 35.0–69.2). These baseline characteristics are summarized in Table 1.

**Table 1:** Baseline characteristics of included patients with AIS^‡^.

**Characteristics**	**Total (*N* = 216)**
Age, years	66 (56–75)
Sex, male	132 (61.1)
**Arterial pressure, mm Hg**	
Systolic	155 (130–175)
Diastolic	95 (85–105)
Body temperature, °C	37.1 (36.6–37.6)
BMI, kg/m^2^	25.5 (24.0–27.1)
**Vascular risk factors**	
Hypertension	155 (71.8)
Hypercholesterolemia	71 (32.9)
Diabetes mellitus	59 (27.3)
Atrial fibrillation	20 (9.3)
Coronary heart disease	18 (8.3)
Family history for stroke	12 (5.6)
Smoking	46 (21.3)
Drinking	44 (20.4)
**Stroke severity at admission** ^†^	
NIHSS score	7 (4–12)
Minor stroke	88 (40.7)
Moderate stroke	67 (31.1)
High stroke	61 (28.2)
DWI lesion size	121
Small	52 (43.0)
Medium	41 (33.9)
Big	28 (23.1)
**Laboratory findings**	
Serum glucose level, mmol/L	5.5 (4.9–6.3)
Serum C–reactive protein level, mg/l	4.1 (3.2–9.5)
Serum HCY level, umol/l	15.1 (11.9–19.5)
Serum IL-33 level, pg/ml	52.6 (35.0–69.2)
**Therapies before admission**	
Antihypertensive	133 (61.6)
Anticoagulant	38 (7.6)
Statins	48 (22.2)
Antiplatelet agents	55 (25.5)
**Acute treatment**	
IV thrombolysis	24 (11.1)
Mechanical thrombectomy	6 (2.8)
IV thrombolysis and/or mechanical thrombectomy	27 (12.5)
**Stroke causative factors**	
Large-vessel occlusive	59 (27.3)
Small-vessel occlusive	51 (23.6)
Cardioembolic	46 (21.3)
Other	15 (6.9)
Unknown	45 (20.8)
Hospital stays, days	11 (7–16)
Hospital cost, CNY	11.500 (8.850–18.100)

^‡^Results were presented as median with interquartile range (IQR) and frequency with percentage.

^†^Stroke clinical severity at admission was dichotomized as minor (NIHSS, ≤ 5), moderate (NIHSS, 6–10), and high (NIHSS, >10) stroke. Lesions were ranked into three sizes to represent typical stroke patterns: (1) small lesion with a volume of < 10 ml, (2) medium lesion of 10–100ml, and (3) large lesion with a volume of more than 100 ml.

BMI, body mass index; IL-33, interleukin-33; IQR, interquartile range; NIHSS, National Institutes of Health Stroke Scale; DWI, diffusion weighted imaging; HCY, Homocysteine; CNY, Chinese Yuan.

### IL-33 and stroke characteristics

**Figure 2:**
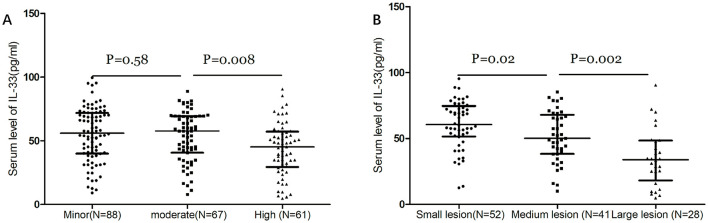
Dot plots of the serum level of IL-33 according to the stroke severity. **(A)** Dot plots of serum levels of IL-33 according to the severity of stroke as defined by the NIHSS score. Stroke patients were divided into 3 groups by NIHSS score according to the following standards: minor stroke (NIHSS ≤ 5), moderate (NIHSS, 6–10) and high clinical severity (NIHSS > 10). **(B)** Dot plots of serum levels of IL-33 according to the severity of stroke as defined by lesion size. Stroke patients with diffusion-weighted imaging (DWI) data were divided into 3 groups by lesions: small lesions with a volume of <10 ml; medium lesions of 10–100 ml; and large lesions with a volume of more than 100 ml. Mann–Whitney U Test; All data are medians and interquartile ranges (IQR), with dot plots representing all values.

IL-33 serum levels were found to decrease with increasing stroke severity as defined by NIHSS scores and lesion size. There was a significant negative correlation between NIHSS scores and IL-33 levels (r = −0.257, *P* < 0.001). At admission, 88 patients (40.7%) had minor strokes (NIHSS ≤ 5), 67 (31.1%) had moderate strokes (NIHSS 6–10), and 61 (28.2%) had severe strokes (NIHSS > 10). Interestingly, the median IL-33 level was highest in patients with minor strokes (56.1 pg/ml [IQR, 40.0–71.9]) and moderate strokes (57.8 pg/ml [IQR, 40.7–69.3]), and lowest in severe strokes (45.2 pg/ml [IQR, 29.5–57.2]), with significant differences noted (*p* < 0.01, Figure 2A). Among the 121 patients with available MRI data, IL-33 levels inversely correlated with lesion size (Figure 2B). Median IL-33 levels were 60.6 pg/ml (IQR, 51.5–74.9) for small lesions, 50.2 pg/ml (IQR, 38.4–68.0) for medium lesions, and 34.0 pg/ml (IQR, 18.2–48.5) for large lesions (P < 0.05). Additionally, patients with large artery ischemic strokes exhibited significantly higher median IL-33 levels (47.4 pg/ml [IQR, 30.5–55.1]) compared to those with other ischemic stroke subtypes (54.2 pg/ml [IQR, 37.2–71.3]; *P* = 0.013).

### IL-33 and stroke prognosis

A total of 80 patients (37.0%) experienced an unfavorable functional outcome, defined as a modified Rankin Scale (mRS) score of 3–6, and 30 patients died within 90 days, resulting in a mortality rate of 13.9%.

**Figure 3:**
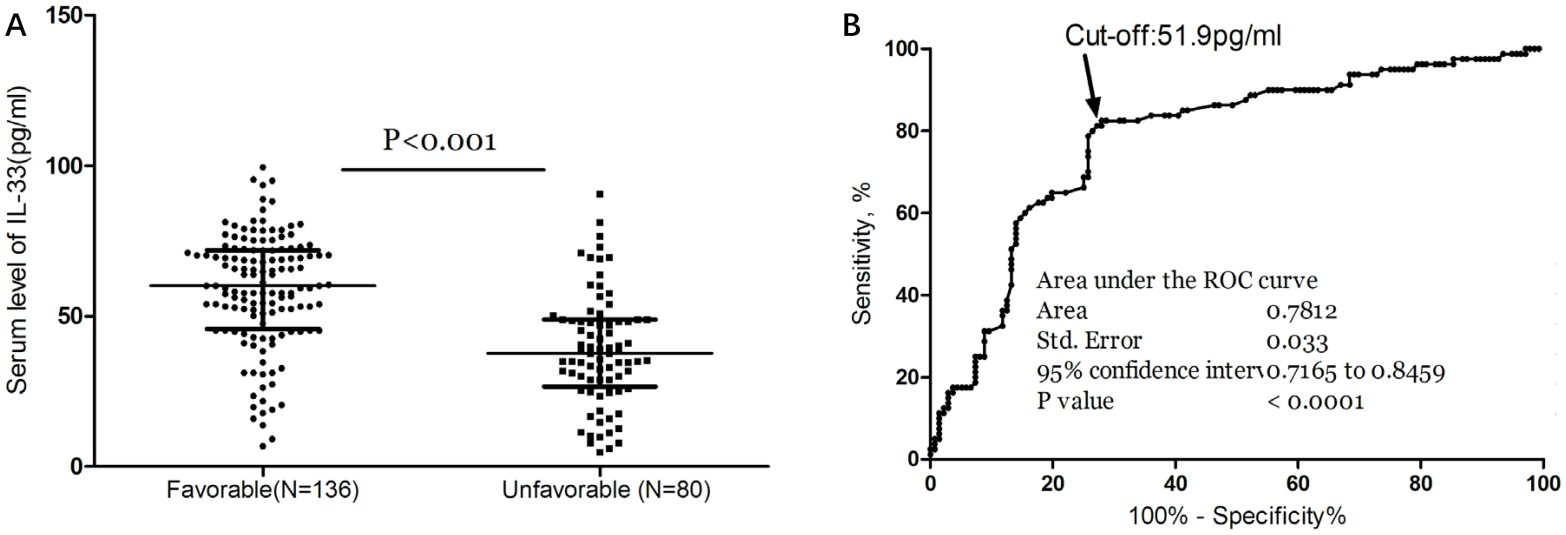
Serum IL-33 and stroke functional outcome. **(A)** IL-33 levels in stroke patients with favorable and unfavorable functional outcome; Mann–Whitney U Test; All data are medians and interquartile ranges (IQR), with dot plots representing all values. **(B)** Receiver operator characteristic curve demonstrating sensitivity as a function of 1-specificity for predicting the functional outcome within 3 months based on serum level of IL-33.

As depicted in Figure 3A, median IL-33 levels were significantly higher in patients with favorable outcomes compared to those with unfavorable outcomes (60.0 pg/ml [IQR, 45.8–71.9] vs. 37.6 pg/ml [IQR, 26.6–48.9]; P < 0.001). In the multivariate logistic regression analysis, lower IL-33 levels were associated with a higher rate of unfavorable outcomes (reduced vs. normal: 55.6% vs. 18.5%; absolute difference, 29.2% [95% CI, 24.5% to 34.4%]; OR, 3.19 [95% CI, 1.72 to 5.91]) (Table 2). Among the subgroup of 121 patients who underwent MRI evaluations, reduced IL-33 remained an independent predictor of poor outcome (OR, 3.57 [95% CI, 1.82–6.63]; *P* < 0.001), after adjusting for lesion size (OR, 1.75 [95% CI, 1.25–2.32]; *P* = 0.003) and NIHSS score (OR, 1.15 [95% CI, 1.05–1.24]; *P* < 0.001).

**Table 2:** Multivariate logistic regression analysis of predictors of stroke prognosis^‡^.

**Predictor**	**OR**	**95%CI**	***P***
**Multivariate analysis for unfavorable functional outcome** ^†^			
IL-33 (reduced vs. normal)	3.19	1.72–5.91	< 0.001
Age (increase per unit)	1.09	1.03–1.15	< 0.001
Stroke severity (NIHSS increase per unit)	1.21	1.13–1.29	< 0.001
HCY (increase per unit)	1.18	1.05–1.32	0.008
CRP (increase per unit)	1.07	1.01–1.15	0.012
Acute stroke treatment (yes vs. no)	0.29	0.15–0.53	< 0.001
**Multivariate analysis for mortality**			
IL-33 (reduced vs. normal)	4.12	1.38–12.31	< 0.001
Age (increase per unit)	1.08	1.02–1.16	0.003
Stroke severity (NIHSS increase per unit)	1.20	1.13–1.30	< 0.001
HCY (increase per unit)	1.15	1.03–1.30	0.015
CRP (increase per unit)	1.09	1.02–1.17	0.010
Acute stroke treatment (yes vs. no)	0.25	0.12–0.59	< 0.001

^‡^Multivariable model included all of the following variables: age, gender, BMI, atrial fibrillation, diabetes, hypertension, NIHSS at admission, stroke subtype, acute stroke treatment, and serum levels of IL-33, CRP, and HCY.

^†^Unfavorable functional outcome of stroke patients was defined as an mRS score of 3–6 points.

BMI, body mass index; IL-33, interleukin-33; NIHSS, National Institutes of Health Stroke Scale; HCY, Homocysteine; CRP, C–reactive protein.

The ROC curve identified an optimal cut-off of IL-33 serum level at 51.9 pg/ml for predicting unfavorable outcomes, with a sensitivity of 82.5% and a specificity of 72.1% (Figure 3B). This threshold yielded an AUC of 0.78 (95% CI, 0.72–0.85), demonstrating significantly greater discriminatory ability compared to age, sex, homocysteine (HCY), and C-reactive protein (CRP), and comparable to the NIHSS score (Table 3). Combining IL-33 with the NIHSS score improved the AUC to 0.84 (95% CI, 0.79–0.90; P < 0.001), with this enhancement confirmed through internal 5-fold cross-validation yielding an average AUC of 0.77 (standard error, 0.034) for NIHSS alone and 0.84 (0.025) for the combined model—a difference of 0.07 (0.008).

**Table 3:** Receiver operating characteristic analysis of predictors of stroke prognosis.

**Predictor**	**AUC**	**95%CI**	***P***
**Prediction of unfavorable functional outcome**			
IL-33	0.78	0.72–0.85	-
NIHSS	0.77	0.71–0.83	0.73
Age	0.71	0.64–0.78	0.005
HCY	0.69	0.61–0.76	0.001
CRP	0.66	0.59–0.74	< 0.001
Combined score (NIHSS/IL-33)	0.84	0.79–0.90	0.007
**Prediction of mortality**			
IL-33	0.82	0.73–0.90	-
NIHSS	0.84	0.77–0.93	0.18
Age	0.74	0.67–0.82	< 0.001
HCY	0.64	0.56–0.76	< 0.001
CRP	0.65	0.57–0.76	< 0.001
Combined score (NIHSS/IL-33)	0.88	0.83–0.94	0.006

IL-33, interleukin-33; NIHSS, National Institutes of Health Stroke Scale; HCY, Homocysteine; CRP, C–reactive protein; AUC, area under the curve; CI, confidence interval.

**Figure 4:**
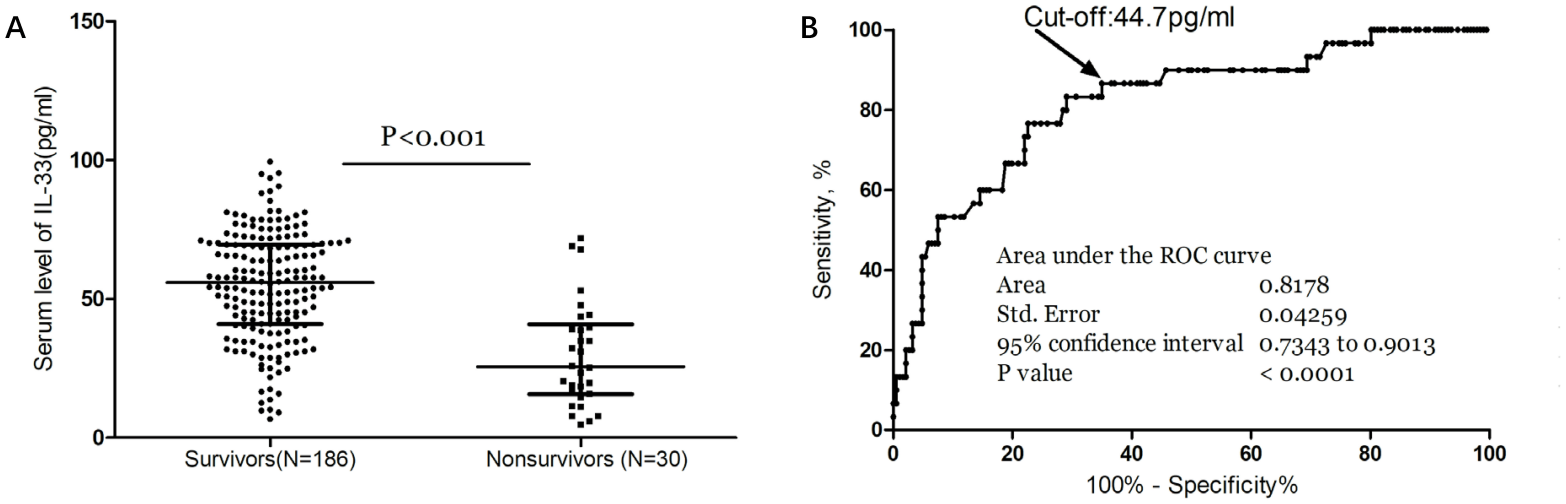
Serum IL-33 and stroke mortality. **(A)** IL-33 levels in survivors and nonsurvivors of stroke; Mann–Whitney U Test; All data are medians and interquartile ranges (IQR), with dot plots representing all values. **(B)** Receiver operator characteristic curve demonstrating sensitivity as a function of 1-specificity for predicting the mortality within 3 months based on serum level of IL-33.

Moreover, median IL-33 levels were significantly higher in survivors compared to non-survivors (56.4 [IQR, 42.2–69.9] vs. 25.7 [IQR, 15.6–38.9] pg/ml; *P* < 0.001; Figure 4A). In multivariate logistic regression analysis, lower IL-33 levels were associated with a higher mortality rate (reduced vs. normal: 24.1% vs. 3.7%; absolute difference, 15.8% [95% CI, 13.1%−18.3%]; OR, 4.12 [95% CI, 1.38–12.31]) (Table 2). Among the 121 patients who underwent MRI evaluations, reduced IL-33 levels independently predicted mortality, with an OR of 4.63 (95% CI, 1.18–13.15; *P* = 0.002) after adjusting for lesion size (OR, 1.88 [95% CI, 1.35–2.76]; *P* < 0.001) and NIHSS score (OR, 1.19 [95% CI, 1.03–1.34]; *P* < 0.001).

**Figure 5:**
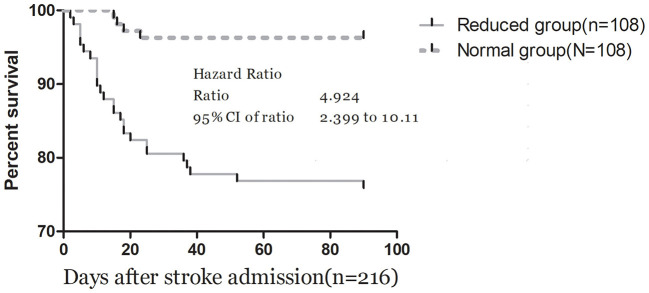
Kaplan–Meier survival based on IL-33 median. Time to death was analyzed by Kaplan–Meier curves based on IL-33 median. Patients in the reduced group (≤52.6 pg/ml) had an increased risk of death compared with patients in the normal group (*P* < 0.001).

The ROC curve analysis determined that the optimal cut-off value for IL-33 serum level in predicting mortality was 44.7 pg/ml, achieving a sensitivity of 83.3% and a specificity of 71.0% (Figure 4B). This resulted in an AUC of 0.82 (95% CI, 0.73–0.90), indicating that IL-33 has significantly greater discriminatory ability compared to age, sex, homocysteine (HCY), and C-reactive protein (CRP), and comparable to the NIHSS score (Table 3). Furthermore, combining IL-33 with the NIHSS score improved the AUC to 0.88 (95% CI, 0.83–0.94; *P* < 0.001), with this enhancement supported by an internal 5-fold cross-validation showing an average AUC of 0.84 (standard error, 0.041) for the NIHSS alone and 0.88 (0.035) for the combined model, a difference of 0.04 (0.006). Kaplan–Meier survival curves, stratified by the median IL-33 level (reduced vs. normal), illustrated that patients in the reduced group (≤ 52.6 pg/ml) had a significantly higher risk of death (*P* < 0.001) (Figure 5).

The association between IL-33 and stroke prognosis was analyzed in a subgroup excluding patients who received acute reperfusion therapy. Among the 75 patients (39.7%) who experienced an unfavorable functional outcome, 29 died within 90 days, resulting in a mortality rate of 15.3%. In multivariate logistic regression analysis, lower IL-33 levels (reduced vs. normal) were linked to a higher rate of unfavorable outcomes (OR, 3.24; 95% CI, 1.66–5.99) and mortality (OR, 4.18; 95% CI, 1.25–12.42).

## Discussion

In this prospective observational study, we found that low serum IL-33 levels are independently associated with greater clinical severity at admission and poorer prognosis at follow-up. IL-33 serves as an independent prognostic marker for both functional outcomes and mortality in patients with ischemic stroke, providing significant additional predictive value beyond the NIHSS clinical score. Furthermore, the prognostic accuracy of IL-33 in stroke patients surpasses that of other commonly measured laboratory parameters and clinical measures.

In previous research, Li et al. (30) identified IL-33 as an independent predictor of functional outcomes in ischemic stroke, with an adjusted OR of 0.932 (95% CI, 0.882–0.986), highlighting its role as a protective prognostic factor. Additional studies support the significance of low IL-33 levels in predicting long-term outcomes in patients with first-ever acute ischemic stroke, with one such study noting a closely related adjusted hazard ratio of 0.979 (95% CI, 0.961–0.997, P = 0.025) for recurrent ischemic stroke (31). Moreover, a concentration of IL-33 ≤ 71.85 ng/L was independently predictive of post-stroke depression, as shown by a multivariate logistic regression analysis (95% CI, 1.129–7.515, *P* = 0.027) (32). Another study reported that low IL-33 levels, alongside increased sST2 levels, predict mortality risk in critically ill patients (33). Consistent with these findings, our research also demonstrated that lower serum IL-33 levels are associated with larger infarction volumes and greater stroke severity in AIS patients, corroborating evidence from earlier studies (30). Compared to previous studies (30–32), this research provides new insights into the prognostic value of low serum IL-33 levels in acute ischemic stroke (AIS) by demonstrating that lower IL-33 levels are associated with increased stroke severity, worse functional outcomes, and higher mortality rates. Unlike earlier studies (30–32), this research adds a combined analysis with NIHSS scores to improve the prognostic accuracy of IL-33, showing its enhanced predictive power for unfavorable outcomes and mortality. It also uniquely evaluates the serum IL-33 levels as a biomarker in a cohort of Chinese patients, contributing to the regional understanding of stroke prognosis.

These studies, together with our findings, support the hypothesis that IL-33 might has a protective role in cerebral stroke. However, further clinical research is necessary to determine whether IL-33 supplementation could improve stroke prognosis. Notably, one study demonstrated that IL-33 promotes a Th2 response while suppressing a Th17 response following middle cerebral artery occlusion (MCAO), indicating a protective mechanism (34). Another study observed that IL-33 administration not only increased levels of interleukin-4 in the brain and periphery but also conferred protection in a mouse model of cerebral stroke (35). Beyond neuroprotection, IL-33 has been identified as potentially therapeutic in other conditions. Veeraveedu et al. (36) highlighted its cardioprotective role in stressed myocardium, suggesting its utility in treating nonischemic heart failure. Additionally, IL-33 has been shown to prevent cardiomyocyte apoptosis, improve cardiac function and survival post-myocardial infarction via ST2 signaling (13), and reduce mortality in experimental sepsis by enhancing neutrophil influx to infection sites (37). Furthermore, Martínez-Martínez et al. (38) found that IL-33 could attenuate metabolic disorders associated with aldosterone excess and inhibit aldosterone-induced adipocyte differentiation and inflammation. Another study indicated IL-33's protective role in mitigating adipose tissue inflammation during obesity (39).

## Limitations

Several limitations of this study warrant consideration. First, its observational nature precludes determination of causality, and residual confounding factors such as poorer health status may influence the findings. Second, IL-33 levels were measured only once upon admission, which may not accurately reflect chronic levels. Third, although IL-33/ST2 signaling plays a dual role in various central nervous system (18) and cardiovascular diseases (40, 41), this study did not measure ST2 levels, which limits our ability to fully assess the role of IL-33/ST2 signaling in stroke prognosis. Fourth, although one study identified a significant association between the IL-33 gene polymorphism rs4742170 and ischemic stroke development (42), this study did not examine genetic variations. Fifth, since this study focuses primarily on stroke patients, I did not analyze IL-33 concentrations in a healthy population. However, previous research has shown that serum IL-33 levels are significantly higher (*P* < 0.001) in patients with AIS [57.68 ng/L (IQR, 44.95–76.73)] compared to healthy controls [47.48 ng/L (IQR, 38.67–53.78)] (30). Lastly, the study cohort consisted solely of Chinese patients recruited from a single hospital, which may introduce selection bias. Validation with an independent cohort would enhance the generalizability and validity of the results.

## Conclusion

In conclusion, our findings suggest that low IL-33 levels are linked to greater stroke severity and poorer outcomes, reinforcing the potential of IL-33 as a biomarker for predicting adverse prognosis following acute ischemic stroke. Further research is needed to determine whether IL-33 supplementation could benefit patients with low serum levels, thereby potentially improving stroke outcomes.

## Data Availability

The raw data supporting the conclusions of this article will be made available by the authors, without undue reservation.
